# *“We Copy to Join in, to Not Be Lonely”*: Adolescents in Special Education Reflect on Using Dramatic Imitation in Group Dramatherapy to Enhance Relational Connection and Belonging

**DOI:** 10.3389/fpsyg.2020.588650

**Published:** 2020-12-17

**Authors:** Amanda Musicka-Williams

**Affiliations:** Creative Arts and Music Therapy Research Unit, Faculty of Fine Arts and Music, The University of Melbourne, Melbourne, VIC, Australia

**Keywords:** dramatic imitation, adolescents, special education, belonging, dramatherapy, co-created research

## Abstract

This paper focuses on doctoral research which explored relationships and interpersonal learning through group dramatherapy and creative interviewing with adolescents in special education. A constructivist grounded theory study, positioning adolescents with intellectual/developmental disabilities as experts of their own relational experiences, revealed a tendency to *“copy others.”* The final grounded theory presented *“copying”* as a tool which participants consciously employed “*to play with,” “learn from,”* and *“join in with”* others. Commonly experiencing social ostracism, participants reflected awareness of their tendency to *“copy others”* being underpinned by a need to belong. Belonging was therefore expressed as the ultimate therapeutic experience participants wished to have. Participant responses which link dramatic imitation to a self-identified tendency *“to copy,”* are discussed with regard to how imitation provides an accessible point of dramatic entry from which adolescents in special education begin to explore new ways of being and inter-relating. Recommendations for how dramatherapists might centralize imitative aspects of the dramatic process to achieve therapeutic intent when working alongside adolescents in special education are discussed with specific focus on creating a space of belonging.

Note on type: Participant quotes extracted from the data are included throughout this article. In order to highlight participant’s contributions quotes are italicized and presented within speech marks.

## Introduction

“Copying? Yeah I do that. I do it a lot.”

“I copy others because it’s important to feel included, to not be left out.”

“In dramatherapy we do a lot of copying. It’s both similar and different to the copying we do elsewhere”

This report discusses key findings from doctoral research exploring relationships and interpersonal learning through group dramatherapy and creative interviewing with adolescents in special education. A constructivist grounded theory study, the research positioned adolescents with intellectual/developmental disabilities as experts of their own relational experiences. Participants engaged in creative interviewing to reflect on their exploration of relational experiences in group dramatherapy. Through dramatic action and verbal reflection participants identified a tendency to *“copy others.”* They described *“copying”* as a source of social learning (Legare and Neilson, 2015; Legare et al., 2015), an act which they engaged in to *“play with*,” *“learn from,”* and *“join in with others.”* This paper limits discussion of the research findings to focus on participant’s insights about their conscious use of dramatic imitation to enhance “belonging.”

Research participants communicated common experiences of social ostracism within peer group/s and the wider community. Research within the creative arts therapies acknowledges that people diagnosed with intellectual/developmental disabilities are commonly stigmatized and socially ostracized (Bailey, 2016; Snow et al., 2017). Participants enacted and reflected upon their tendency to *“copy others,”* acknowledging how such actions were underpinned by a human need to experience belonging (Baumeister and Leary, 1995). Belonging was expressed as the ultimate therapeutic goal, for young people who desired *“to be treated as equal”* and perceived as *“just normal teenagers”* who *“want to join in and be included, just like everybody else.”* In this paper, participant reflections on *“copying”* are linked to a discussion of dramatic imitation, which explores how embodied and imitative aspects of dramatherapy were familiar to participants. This familiarity provided easy entrance into imaginal play, an extended experience of self in relationship to others (Tytherleigh and Karkou, 2010) and ultimately the means by which participants enhanced their desire for belonging.

## Design and Method

This research adopted a view that, “qualitative research inherently invites creativity and the use of innovative methods to gain an insight into the participant’s world” (Halcomb, 2016: 6). A mixed methodological design incorporated a constructivist grounded theory approach to research which was influenced by inclusive (Walmsley et al., 2018) and arts informed (Cole and Knowles, 2011; Leavy, 2015) research practices. Constructivist grounded theory was chosen as a systematic approach to data collection and analysis (Engward, 2013; Charmaz, 2014) which enabled participants’ voices to be centralized within the generation of new theory ([opethcitep]B7,B8,B9[clothcitep]Charmaz, 2011,2012,2014). The aims of inclusive research supported a desire to challenge prevailing assumptions that people with intellectual disabilities cannot engage with/have little to contribute to research (Walmsley et al., 2018). The research focused on *“relationships with others”* as a participant chosen topic, reflecting the aim of inclusive research to engage with research topics relevant to participant’s lives (Walmsley et al., 2018).

Arts informed research provided the inspiration for including dramatherapy techniques as data collection methods. Amalgamation of these different research approaches aimed to provide participants with the means and motivation to actively engage with research. Undertaken within a place of professional practice, the research was further defined as practitioner research, aiming to address the needs of participants in relation to a specific context (Jones, 2010; Hay-Smith et al., 2016; Miller, 2017). Outcomes were intended to inform the future practice of dramatherapy in special education.

### Context and Familiarity

The research was conducted at Port Phillip Specialist School, in Melbourne, Australia. The school supports students with intellectual/developmental disabilities to achieve learning outcomes related to the development of independent living skills. It adopts a unique curriculum in which creative arts programs are a source of experiential learning. At the time of data collection, the researcher had been the dramatherapist for over 12 years. Therefore, ethical concerns associated with the dual role of the practitioner researcher needed to be addressed. This included management of potential role confusion for participants, with regard to the researcher’s role, and a need to vigilantly monitor researcher assumptions (Hay-Smith et al., 2016; Hiller and Vears, 2016).

Strengths in undertaking practitioner research in this context were also perceived. Prior establishment of trust and rapport served to ease participant anxiety about engaging with unfamiliar research processes (McVilly et al., 2006). While previous engagement with dramatherapy, either individually or within group-work meant that participants were confident in using drama as a means of self-expression. Familiarity with the researcher and dramatherapy process assisted an overall aim to promote a position of mutuality and position participants as co-creators of knowledge (Charmaz, 2014; Huss, 2017).

### Participants

The participants consisted of 15 adolescents from Port Phillip Specialist School. Eligibility to attend a special school is dependent upon a formal diagnosis of intellectual disability (ID) as assessed by a registered psychologist in accordance with criteria for ID outlined in the DSMIV. This assessment includes an IQ and adaptive life skills test using the Vineland. Tests are undertaken at school entry and reviewed at key developmental stages. Test results indicated that 13 of the participants were diagnosed with mild (IQ range 52–69) to moderate (IQ 36–51) ID. The remaining two participants scored above 70 in their IQ test during their final adolescent review, while maintaining a below 70 score in adaptive skills. This result enabled their continued attendance at the school. Participants ranged from 15 to 18 years of age and consisted of seven females and eight males. They represented diverse familial, economic, and cultural backgrounds. Non-verbal students or those whose IQ was below the mild-moderate range were excluded due to data collection by interviews.

### Consent Process

Parents/caregivers were required to give written consent for participants under their care. However, the adolescents had final right of assent. The assent process was run as a creative workshop, with experiential activities intended to enhance participant understanding of what was required if they chose to engage with the research (Musicka-Williams, 2018). Recognizing the potential for participants to experience ongoing challenges with comprehending research tasks and aims, an ongoing process of assent was negotiated (Ericsson and Boyd, 2017).

### Ethical Approval

Ethical approval to conduct the research was provided by the University of Melbourne’s Ethics Committee and the Victorian Education Department.

### Research Aims/Focus

The research focused on two inter-related aims of inquiry:

1. To explore participant’s experiences and perceptions of key relationships in their lives through group dramatherapy and creative interviewing.
2. To reflect with participants on the use of dramatherapy to promote experiential learning (Butler, 2017), with specific focus on the development of relational skills.

The inter-related focus acknowledged the ways in which therapeutic goals are often intertwined with learning objectives when dramatherapy is undertaken in educational settings (Holmwood, 2014; Frydman and Mayor, 2019). Subsequent data collection supported the decision to maintain this dual focus of inquiry, as participant’s linked experiences in dramatherapy to personal learning outcomes.

### Group–Work Activities

To explore their *“important relationships”* and related relational themes, participants engaged in a variety of dramatherapy practices including:

• Dramatic engagement with role theory and exploration of one’s own personal role repertoire ([opetwcitep]B34,B35[clotwcitep]Landy, 1993,2009; Landy and Butler, 2012).
• Role play, improvization, and tableaux depicting real life relational experiences.
• Story making and story-telling incorporating art-making and projected play to explore relational themes.
• Structured games, movement tasks, and dramatic problem solving scenarios linked to relational experiences.
• Reflective practice incorporating artwork, dramatic gesture, group discussion, and the use of a spectrogram to acknowledge commonalities/differences (Dunne, 2012).

## Data Generation Through Creative Interviewing

Acknowledging participant’s reflections that, *“sometimes showing is easier than telling,”* and influenced by creative interviewing, where artistic techniques are incorporated into qualitative interviewing (Nind and Vinha, 2016), dramatherapy methods were embedded into a semistructured interview process. This approach was further influenced by “dynamic re-enactment,” an interviewing technique employed in marketing research, where the researcher “watches someone do something and then asks questions” (Collier, 1993: 35). Incorporating dramatic action aimed to elicit and extend participant’s capacity for verbal insight, overcoming a common participant experience in which *“thoughts become stuck”* when asked direct questions.

During interviews participants reflected on video recordings of the dramatherapy sessions. For some participants this was helpful, enabling them to respond to questions about engagement in group dramatherapy and the relational experiences explored, without pressure to recall events. For others, video recordings were difficult to engage with. Participants with impaired hearing, sight, or sensory processing had difficulty following video footage. Incurring these responses confirmed the need to offer multi-modal tools of response.

Participants were offered the choice to verbalize or dramatize responses to research inquiry. Various dramatic forms were used to respond including; role-play, improvization, tableaux, puppetry, projected play, and story-making through art. Familiarity in engaging with dramatic techniques appeared to ease participants’ flow of expression, resulting in spontaneous dialog accompanying dramatic actions/renewed capacity to respond to inquiry while engaged in dramatic play.

Each participant was interviewed twice during the 16 week dramatherapy program. The primary researcher conducted dramatherapy sessions and interviews. Participants were chosen to attend interviews following a demonstration of personal insight in a previous dramatherapy session. The average length of interview was 40 min. All interviews were audio recorded for analysis. Participants were able to end interviews upon request.

### Data Analysis

An iterative and systematic process of constant comparative data analysis (Engward, 2013) congruent with coding practices of constructivist grounded theory (Charmaz, 2014) was undertaken. Data were coded according to identification of “conceptual re-occurrences and similarities in the patterns of participant’s experiences” (Birks and Mills, 2015: 176). Open coding and cross comparison of data identified reoccurrences as consistent categories (Birt et al., 2016). Categories then informed the direction of inquiry taken in follow up interviews which underwent the same coded analysis (Charmaz, 2014). Alongside consistencies within the data, the emergence of new categories were acknowledged and incorporated into the next stage of analysis. Each analysis stage was reviewed with academic supervisors.

Due to incorporation of dramatherapy techniques within the interviews, coding was divided into three distinct types reflecting different forms of expression chosen by participants.

1. Word codes: identification of themes related to the interpreted meanings of words.
2. Action codes: descriptive code for actions undertaken by participants.
3. Delivery codes: code describing the way participants spoke.

In final stages of analysis, all codes were treated equally and merged according to a thematic interpretation. Mind maps identifying connecting codes were used to review data in new formats (Konecki, 2011; Chandrasegaran et al., 2017) consistent with the aims of axial coding. Repetitive cross comparison of data and re-occurring themes identified the final core category and connecting categories (Giles et al., 2016).

The core category of *“copying”* was reflected upon in individual member checking (Birt et al., 2016) where participants confirmed, rejected, or extended previous comments about *“copying others”* through co-created data. Participants extended upon relational maps previously created in dramatherapy, to artistically represent their *“important relationships.”* On these maps, they represented in words and pictures the presence or absence, and purpose of *“copying”* in each of their relationships.

## Results

### Final Grounded Theory

The final grounded theory focused on understanding the purpose behind participant’s tendency to *“copy others.”* It describes *“copying”* as an act of imitative learning which participants engaged in during group dramatherapy and in wider relational/learning contexts. The theory presents *“copying”* as an action that participants consciously employ to; *“play with,” “learn from,”* and *“join in with”* others. These purposes are further defined as participant’s self-identified therapeutic objectives.

**FIGURE 1:**
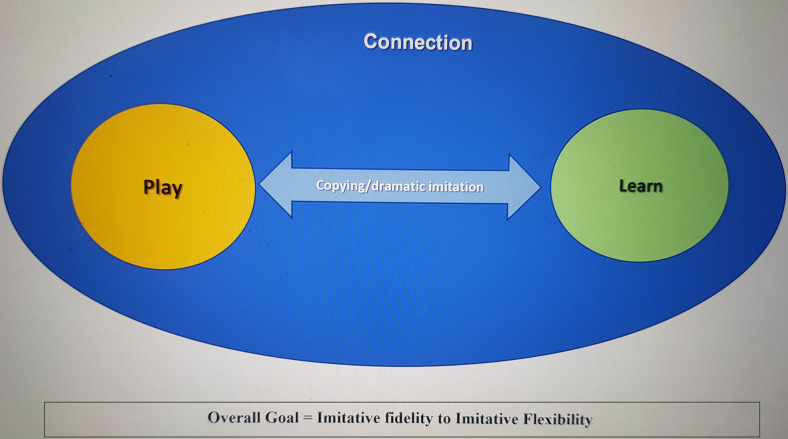
Accessible representation of the grounded theory.

Figure 1 presents a visual summation of the grounded theory and the inter-relating functional properties/purposes of *“copying others.”* It was constructed as a simple visual to enable participants to identify, engage with, and reflect upon their ideas.

### Key Theoretical Concepts

Participants described the phenomenon of *“copying”* by different terms; *“following,” “mimicking,” “mirroring,” “repeating,” “doing what the others do,” “imitating,”* or *“doing charades.”* Furthermore, they enacted “*copying”* in ways which demonstrated conscious choice in dramatic style. Some *“copying”* was precise and naturalistic, representing a direct replication of a person/events. Other presentations were stylized and innovative, engaging participants in a selective process of what original elements were imitated. Participants chosen terms therefore indicated both the dramatic style presented and the intended purpose for “*copying others.”* Psychological and educational theory related to imitative learning provides a theoretical framework from which to construct and explain consistently identified purposes of *“copying others”* which are presented as central concepts of the grounded theory.

• Imitative learning is a social learning practice where a learner acquires new behaviors/skills by imitating others (Kale-Kruger, 2011; Over and Carpenter, 2012). Participants described this as “*Watching and copying so you know what to do”* or *“Seeing and doing.” “Copying others”* was perceived as *“the easiest way to learn.”*

• High fidelity imitation occurs when the learner completes a direct replication of skill sets modeled through action sequences (Legare and Neilson, 2015; Legare et al., 2015). Participants referred to this as, *“Straight copying.”*

• Over imitation occurs when learners imitate all detail of actions, regardless of whether those details are purposeful in achieving an end goal (Legare and Neilson, 2015). Participants described this as, *“Being a copy-cat.”*

• Imitative flexibility refers to the learner’s capacity to demonstrate innovation when reproducing skills/behaviors observed in others (Legare and Neilson, 2015; Legare et al., 2015). Participants referred to this as, *“Copying something and then finding a way to make it your own.”*

### Enhancing Imitative Flexibility

Imitation is presented as central to the participants’ experience of therapeutic change. Participants reflected that, *“In dramatherapy it’s easy to join in and learn because there’s lot of copying.”* They viewed dramatic imitation as a kind of *“copying,”* where *“we copy and act out things from real life.”* Participants further confessed that *“straight copying”* of others was limited and *“annoying”* and resulted in being *“discluded.”* To avoid this participants recommended, *“You can start with copying, but it can’t be everything. Eventually you have to find a way to make it your own.”*

The overall therapeutic goal identified within the grounded theory is to use dramatic imitation to move participants from a state of play in which they engage in acts of high fidelity imitation to an exploration of their own capacity for imitative flexibility. A participant who could innovate upon the imitation was seen as *“a leader*,” someone that *“everyone wants to be like,”* a role which secured inclusion and belonging.

## Discussion

### Psychological Benefits of Imitation

Participants’ reflections on their human tendency to imitate (Garrels, 2011; Over and Carpenter, 2012) identify psychological benefits associated with imitating others. Understanding of these benefits was deepened by viewing participant’s copying behaviors through reference to psychological, developmental, and educational theories. Relevant theories were chosen for the ease in which concepts could be translated into participant’s own language. Some of the specific benefits of *“copying others”* cited by participants and expanded upon through reference to theory are presented for discussion.

### Imitation and Belonging

“We copy to join in, to not be lonely”

Participants ultimate goal in *“copying others”* both within and outside of group dramatherapy was to create a sense of commonality and belonging. Baumeister and Leary’s theory of belonging proposes that humans have a fundamental need to experience connection and belonging. A drive to form, and maintain interpersonal relationships characterized by mutual respect and concern (1995). Participants echoed these ideas, *“I just want everyone to be included…to be treated equal.” “Everybody copies to join in because no one wants to be left out, to be lonely.”* Participants described experiencing connection and belonging within dramatherapy that they did not experience elsewhere. “*In dramatherapy we do lots of copying….It’s good, because it’s easy to join in and everyone gets included.”*

### Imitation and Adolescence

“We do it because we are teenagers, Teenagers copy each other a lot.”

Imitative behavior occurs throughout the human life span, its goals increasing in complexity and related to the specific developmental/psychological pursuits of different life stages (Garrels, 2011). From earliest stages of development, effective reading and imitation of social cues/behaviors enables people to respond to changing social encounters (Jennings, 2011). Understanding the participants’ unique experience of *“copying others”* included consideration of their adolescent life stage.

An adolescent tendency to imitate peers and form tribe-like cultures is thought to serve protective purposes, until a more fixed adult identity is formed (Frankel, 1998). While rallying together in rejection of adult authority, adolescents express a need to belong to a community of their own (Malekoff, 2015). Imitating peers provides opportunity to counterbalance contradictory desires to reject and belong. Through copying each other, they develop their own adolescent culture, performing common behaviors/self-presentations which define their own rules for belonging.

### Imitation and Role Development

“I copy to try something new, be something different.”

Adolescence is a life stage characterized by role-fluidity (Zeal, 2011; Weber and Haen, 2016). *“Copying others”* enabled participants to explore potential roles. Such an exploration is considered beneficial during adolescence, providing a means to avoid becoming stuck within a limited role repertoire (Dunne, 2012), which impacts opportunities to engage in a range of life experiences. However, people diagnosed with intellectual/developmental disabilities are afforded limited social roles due to stigmatization and prevailing societal attitudes about what they can or cannot represent (Zolkowska, 2016). Participants sought to overcome such limitations by *“copying”* the presentations of others.

“We copy the adults so we know how to be one”

“Everyone copies the leader because they want to be like him”

“When we copy we show that we can do things, just like normal teenagers, that we are just as capable.”

In the role method, Landy proposes that dramatherapy provides a dramatic space in which clients draw from, reconstruct, and reflect upon roles presented to them in every day dramas (Landy, 2009; Landy and Butler, 2012; Klees, 2016). Construction of the role method draws upon earlier sociological and dramatic versions of role theory which support the idea that much of an individual’s role repertoire is based upon prior experience of others in that role (Landy, 2009; Landy and Butler, 2012). Participants identified an ability to extend upon roles available to them by imitating and subsequently innovating roles originally performed by others. Dramatic imitation provided an accessible pathway to expand their individual role repertoires.

### Overcoming Social Ostracism

“We are just normal people who want to be included just like everyone else.”

Reflecting on their relational lives participants generally focused on similarities to others rather than differences. They expressed a desire to be viewed via their potential to be *“just like everyone else”* rather than through what they believed was other’s tendency to focus on perceived deficits.

“People think because we go to a special school we can’t really do much. We get stigmatized…. When we copy we show other people what we can do, that we are capable. That we are just normal people like everyone else.”

Participants described social ostracism as a common experience within peer groups and community contexts.

“In our class there are insiders and outsiders…. There’s like an inner circle and an outer circle. It’s kind of like there are kings and queens, and everyone has to follow and do what the kings says…. And some get included and some of us get discluded.”

“I don’t really know many people outside of school. They don’t include me. They look at me funny because they don’t know me and they don’t know what I can do.”

In response to repeated exclusion, participant’s tendency to *“copy others”* may be viewed as a sign of personal resilience, a survival technique, expression of an ultimate desire to belong. Furthermore, within the group dramatherapy, *“copying”* was viewed as an accessible entry point into dramatic play where new, imaginal possibilities enabled access to desired experiences. Participants described group dramatherapy as providing a space for belonging that did not exist in their wider social worlds.

“In dramatherapy everyone is included. Everyone gets their turn, to have their say and people listen. It’s good because it encourages people to change. Everyone is treated equally. That doesn’t usually happen.”

## Recommendations for Practice

### Imitation as a Core Process

The participants identified that the development of their own capacity to imitate and subsequently diverge from that imitation is central to what makes the dramatic experience therapeutic. Identifying dramatic imitation as a core process for achieving therapeutic change invites continued reflection on the core processes of dramatherapy as a widely accepted framework through which dramatherapists understand client change (Jones, 2016). Jones (2016) acknowledges that different clients and contexts re-define what supports client growth and change. Therefore, the core processes remain open to expansion. Future research into dramatherapy change mechanisms might look to re-construct the core processes in ways which acknowledge unique client perspectives.

### Working Therapeutically With Dramatic Imitation

Consciously working with participant’s dual desire to imitate and innovate (Legare and Neilson, 2015) aims to develop a practice and pace which supports adolescents in special education to access their own ways of enabling personal change. By acknowledging the reflections of these participants, and deliberately centralizing the imitative aspect of dramatherapy to therapeutic effect, dramatherapists have opportunity to make the process meaningful to participants who find *“copying”* an accessible pathway to self-development. Dramatic imitation offers the expansive possibilities of imitative flexibility, where *“you start by copying but then you try something different*… *find a way to make it your own.”*

Participants’ ability to traverse from high fidelity imitation to imitative flexibility is dependent upon a session structure which centralizes and scaffolds imitative practice. Dramatic imitation provides a familiar entry point into dramatic play for participants who are self-confessed copiers, while the imaginative aspect offers potential to transform imitative acts into unique self-presentations. The imaginative aspect of dramatherapy serves as a protective factor (Pendzik, 2018) while participants explore extended role repertoires, enabling those who experience themselves as *“stigmatized”* to demonstrate capacity for creative innovation of self. Innovation which, when enacted outside the therapy space, may invite inclusion in wider social/relational experiences.

### Limitations

The grounded theory constructed from this research represents specific experiences of the 15 participants. Further research would need to be undertaken to determine its usefulness in other special educational contexts. Future research might also uncover other reasons why adolescents with intellectual/developmental disabilities imitate others.

Participant inclusion/exclusion criteria relied upon school records for pre-existing diagnosis. No independent testing was conducted to confirm diagnostic scores. It was accepted that participants would present with varying degrees of verbal ability and comprehension, therefore no independent testing of participant’s verbal abilities was undertaken.

Once participant’s tendency to imitate was identified, the research focused on understanding the reasons behind this tendency. No formal assessment of imitative ability was made beyond the comparison of participant’s verbal reflections. Future research could employ a broader range of analytical tools to explore the embodied uniformity/diversity of participants’ imitative presentations.

Limitations to the transferability of this research are recognized with regard to the nature of the study as practitioner research and participants’ familiarity with the researcher and dramatherapy. It is proposed that the integrity of the findings be determined by whether they can be considered “trustworthy and believable in that they reflect participants’, researchers’ and readers’ experience of the phenomena” (Corbin and Strauss, 2015, p. 346). It is also acknowledged that the resultant theory is a construction and one possible interpretation of the data (Charmaz, 2014).

## Conclusion

Research in the creative arts therapies advocates for unique pathways of self-representation to be acknowledged in public and professional discourse (Hadley, 2013), while the dramatic arts offer unique spaces of belonging for people marginalized in wider communal settings (Ineland and Sauer, 2016). Adjusting dramatherapy practice to respond to participants’ self-identified tendency to *“copy others”* offers opportunity to hold space for young people diagnosed with intellectual/developmental disabilities in ways which honor and make accessible an adolescent experience that is at once unique, and *“just like the other teenagers.”* The participants have illuminated how imitation, a phenomenon fundamental to dramatic practice and human interaction (Jennings, 2011; Chasen, 2014), may be central to promoting personal growth through dramatherapy. By playing with this idea of *“copying,”* we offer participants with intellectual/developmental disabilities an accessible way to innovate on real life experience. To enable clients with a tendency toward high fidelity imitation to move toward imitative flexibility (Legare and Neilson, 2015). Playing with dramatic imitation offers pathways *“to play,” “learn from,”* and *“join in with”* others for young people previously *“discluded.”* In offering these experiences, we give opportunity to access the ultimate therapeutic experience; a way to connect and belong.

## Data Availability Statement

The data sets referred to in this brief research report are not readily available in their entirety, to the public due to participants request. Only some data sets were approved for public sharing by participants and constructed into artistic/academic forms which provided participant anonymity. Any requests to review the data sets can be made directly to the author.

## Ethics Statement

The studies involving human participants were reviewed and approved by The University of Melbourne Ethics Committee and The Victorian Education Department Research Ethics. Written informed consent to participate in this study was provided by the participants’ legal guardian/next of kin. Written informed consent was obtained from the minor(s)’ legal guardian/next of kin for the publication of any potentially identifiable images or data included in this article.

## Author Contributions

The author confirms being the sole contributor of this work and has approved it for publication.

## Conflict of Interest

The author declares that the research was conducted in the absence of any commercial or financial relationships that could be construed as a potential conflict of interest.
